# The relationship of interacting immunological components in dengue pathogenesis

**DOI:** 10.1186/1743-422X-6-211

**Published:** 2009-11-27

**Authors:** David G Nielsen

**Affiliations:** 1 Department of Microbiology and Immunology, Tulane University, (1430 Tulane Avenue) SL-38 (New Orleans) Louisiana (70112-2699) USA

## Abstract

The World Health Organization (WHO) estimates that there are over 50 million cases of dengue fever reported annually and approximately 2.5 billion people are at risk. Mild dengue fever presents with headache, fever, rash, myalgia, osteogenic pain, and lethargy. Severe disease can manifest as dengue shock syndrome (DSS) or dengue hemorrhagic fever (DHF). Symptoms of DSS/DHF are leukopenia, low blood volume and pressure encephalitis, cold and sweaty skin, gastrointestinal bleeding, and spontaneous bleeding from gums and nose. Currently, there are no therapeutics available beyond supportive care and untreated complicated dengue fever can have a 50% mortality rate. According to WHO DSS/DHF is the leading cause of childhood mortality in some Asian countries. Dendritic cells are professional antigen presenting cells that are primary targets in a dengue infection. Dengue binds to **D**endritic **C**ell-**S**pecific **I**ntercellular adhesion molecule-3-**G**rabbing **N**on-integrin (DC-SIGN). DC-SIGN has a high affinity for ICAM3 which is expressed in activating T-cells. Previous studies have demonstrated an altered T-cell phenotype expressed in dengue infected patients that could be potentially mediated by dengue-infected DCs.

Dengue is enhanced by three interacting components of the immune system. Dengue begins by infecting dendritic cells which in immature dendritic cells is mediated by DC-SIGN. In mature dendritic cells, antibodies can enhance dengue infection via Fc receptors. Downstream of dendritic cells T-cells become activated and generate the very cytokines implicated in vascular leak and shock in addition to activating effector cells. Both the virus and the antibodies are involved in release of complement and anaphylatoxins which can cause or exacerbate DHF/DSS. These systems are inextricable and strongly associated with dengue pathogenesis.

## Dengue Background and Significance

The Dengue Virus is a member of the family Flaviviridae along with other noted viruses Yellow Fever, West Nile, and Japanese Encephalitis. Dengue is a positive stranded RNA arbovirus transmitted by mosquitoes typically Aedes *aegypti*. Dengue fever has spread from the border lands of Texas to South and Central America, from Africa to the Middle East to Indonesia and Australia. The World Health Organization (WHO) estimates between 50 million and 100 million infections every year all over the world[1]. Dengue fever will often present with fever, rash, headache, and myalgia but can also develop into much more serious cases of Dengue Hemorrhagic Fever and Dengue Shock Syndrome (DHF/DSS). Cases of DHF/DSS are increasing rapidly as the virus increases in geographic range, with approximately 25-37% of symptomatic cases of dengue requiring hospitalization [2]. Case fatality rates for Dengue can be as high as 40-50% in untreated patients [3,4]. The dengue virus has a significant impact on the health of those it infects and represents a burdensome cost to the patient and health infrastructure in places that can ill afford new and varied threats. Patients who acquire the disease the first time (primary infections) are often asymptomatic and will generate immunity to homologous strains of the virus; however, ninety percent of DHF/DSS cases come from a second exposure (secondary infection) to a heterologus strain of dengue[5]. Patients with a secondary heterotypic infection are at least 40-80 times more likely to develop DHF/DSS as patients with a primary infection[6]. The mechanisms by which dengue would cause severe disease are currently being elucidated, but the prevailing literature suggests three interacting components necessary for dengue induced immune enhancement. One component is misregulation of cell mediated immunity. In this context, the cross relationship between B cells and T cells begins with dengue infection of dendritic cells that, in turn, promiscuously activates T cells. T cells during a dengue infection have prolific and cross reactive effector functions in addition to producing copious amounts of cytokines that feature prominently in cases of DHF/DSS. A second component in immune enhancement is Antibody Dependant Enhancement (ADE). Heterologus non-neutralizing antibodies recognize dengue epitopes and enhance infectivity in an Fc dependant manner. Further, antibodies have been implicated in an autoimmune disease which can also exacerbate vascular leak and cytokine production. A third interacting component in immune activation is complement. Many of the key cytokines implicated in the cytokine storm that characterizes DHF/DSS are regulated by Complement proteins and associated anaphylatoxins. These three systems both interact and reinforce each other to create a potentially life threatening situation during a Dengue infection.

### Antibodies

Antibody Dependent Enhancement (ADE) has been proposed to be a mechanism by which the immune system may enhance viral pathogenesis[7]. When monkeys were passively immunized concurrently with a viral infection they developed 15 fold higher viral titers than monkeys infected without IgG supplement[8]. However, our understanding of this disease is severely limited by appropriate animal models. Animal models can support viral propagation, but do not exhibit illness unless severely immunocompromised. Epidemiological evidence in Hawaii, Cuba, and Thailand[9] shows populations with previous exposure to the dengue virus are at an increased risk for DHF/DSS. Also infants born to dengue immune mothers were shown to be at an increased risk for DHF/DSS[10]. It's not clear how antibodies enhance viral infection. One hypothesis suggests that non-neutralizing antibodies direct active virions to permissive cells in the immune system[11]. There is no "classical" enhancing antibody since all antibodies will enhance the virus at non-neutralizing concentrations[12]. The Fc receptor (FcR) family is a key component in the ADE pathogenesis model. Fc receptors are found on most phagocytes including dendritic cells and macrophages. The FcR functions as a multisubunit complex that typically binds to IgG and is composed of an α chain for domain recognition, an ITAM (immunoreceptor tyrosine based activation motif), and a γ chain that is responsible for signal transduction. It is thought that IgM does not play a direct role in ADE and instead contributes to disease pathogenesis through activation of complement receptors[13]. IgM antibody enhancement was abrogated when C3R is blocked[14]. A hypothesis suggesting that both IgG and IgM mediate viral enhancement implies that the mechanism for ADE is multivariable. FcR signaling pathways generally leads to activation of the immune cell, though under certain circumstances FcR can lead to immune modulation[15]. The normal interaction of virus with antibody generally leads to neutralization. However, in heterotypic dengue virus infections the antibodies are non-neutralizing and lead to enhancement. Two cell lines expressing either FcγRIA or FcγRIIA have been used to demonstrate that immune complexes can enhance virus infectivity in an FcR mediated fashion. FcγRIA is found exclusively on macrophages and dendritic cells and preferentially binds monomeric IgG, while FcγRIIA is more broadly distributed and preferentially binds immune complexes. When exposed to the immune complexes containing the virus, both cell lines showed enhanced infectivity. However, when the signaling capacity of the Fc Receptor was abrogated, phagocytosis is reduced but enhancement is not affected in FcγRIIA. In the FcγRIA cell line, both the phagocytosis and the immune enhancement are reduced with abrogated cell signaling. The disparity is not yet understood. It does suggest that viral entry and immune enhancement can be mediated by more than a single mechanism. In a different study, three cell types have been used to demonstrate enhancement[16]. U9357 cells which express both FcγRIIA and FcγRI have similar antibody-dependent enhancement capabilities as K562 cells that express just FcγRII. However, the cell type Raji-1 which displays DC-SIGN instead of the Fc receptor showed high viral titers but no antibody enhancement.

### Dendritic Cells

The putative receptor and initial target cell for the virus is DC-SIGN (**D**endritic **C**ell-**S**pecific **I**ntercellular adhesion molecule-3 (ICAM3)-**G**rabbing **N**on-integrin) (CD209) on dendritic cells [17-19]. Dendritic cells are considered crucial to fighting viral infections because of their ability to acquire and display viral antigens that would otherwise evade the immune system. Dendritic cells affect the dengue virus in two ways. Immature dendritic cells express high levels of DC-SIGN which facilitates initial viral binding and entry. While mature dendritic cells do not posses high levels of DC-SIGN, they do facilitate ADE via FcγIIa and FcγIIb receptors. This effect was most prominent with serum dilutions ranging from 1:640 to 1:2,560 with complete neutralization at 1:10. ADE in dendritic cells can increase viral RNA production by over 100-fold making dendritic cells potent components in dengue pathogenesis[20]. Infected dendritic cells also contribute to vascular leak through the production of matrix metalloproteinases (MMPs). MMP-2, MMP-13, and MMP-9 were all dramatically increased in immature dendritic cells infected with DENV2. As a result cell-cell adhesion in cells co-cultured with infected dendritic cells was reduced, there were changes in cell morphology and actin cytoskeleton, and a decrease in PECAM 1 VE-cadherin expression[21].

DC-SIGN has a high affinity to the ICAM3 molecules expressed on T-cells with a complicated system of cross talk that can lead to a variety of outcomes[22]. To become activated, T-cells go through a time consuming and multiphase process that lasts anywhere from 6-24 hours. Adhesion molecules such as ICAM1 and ICAM3 are critical molecules generated by the T-cell during either phase and can bind to the adhesion molecules of DCs particularly DC-SIGN which is a known target of dengue. These molecules are necessary to form a stable synapse between the DC and T-cell[23]. T-cells, in turn, promote further maturation with the expression of CD40L. Further stimulation by cytokines such as TNFα, IFNγ, IL-6, and others can rapidly promote the maturation and sensitivity of dendritic cells. In contrast, simulation of dendritic cells with IL-10 and other anti-inflammatory cytokines promotes a regulatory phenotype for DCs[24]. Regulatory dendritic cells have been shown to attenuate the immune response and promote tolerance in a way analogous to T-regulatory cells. DCs can also activate B-cells through co-stimulation of CD40, IL-6, and IL-12. The crux of DC interaction is in two places: DC maturation and T-cell synapse. Either point represents a potential target for dengue virus immune evasion. Should DCs fail to mature properly, they will not only fail to stimulate T-cells but they may induce tolerance. The DC-T-cell interaction is highly coordinated and disruption of the DC-T-cell synapse could promote dengue pathogenesis. Dengue-specific memory T-cells undergo simultaneous proliferation and apoptosis during a heterotypic infection. The end result is a less efficient and less specific T-cell response. The mechanism for this is unknown but given the intimacy between DCs and T-cells this represents a potentially productive field of research.

## The role of T cells in a dengue infection

There is a clear consensus in the literature about activation of cross-reactive memory T-cells, independent of antibody enhancement, being a pivotal moment in the disease process. As compelling as ADE may be, it can not fully describe a complete picture of dengue pathogenesis such as, intense cytokine storm[25], tissue re-modeling[26], and effector cell activation[27]. The misregulation of T-cells centers around the idea of Original Antigenic Sin, or that a secondary heterotypic dengue infection can stimulate cross-reacting, low affinity T-cells. Activation of effector T-cells and secretion of cytokines define a key development in course of disease associated with dengue virus infection. Four patient studies done in Vietnam[28], India[29], Cuba[30], and Brazil[31] all showed increases in INFγ, TNFα, IL-10, IL-1, IL-6, IL-8, and MCP1 amongst a variety of other cytokines. *In vitro *studies, IFNγ, IL-6, TNFα, and RANTES upregulation also have been posited as important events in dengue pathogenesis[32]. A review of dengue-associated cytokines listed 15 different cytokines modulated by the disease. In short, these cytokines are consistent with widespread T-cell involvement. In particular IFN and TNFα were strongly associated with disease severity and correlate well with T-cell activation. In addition to increases in cytokine levels cellular markers for T-cell activation, CD69, CD38, and CCR7 have been shown to be increased in dengue infection[33] and IFNγ secretion by dengue specific T-cells has been shown to upregulate the number of Fcγ receptors. These receptors also play a noted role in Antibody dependant enhancement[34].

CD8+ cells have been shown to be important in helping control early viral infection;[35] but, the intense proliferation of CD8+ cells can also be implicated in dengue pathogenesis[36]. In tetramer staining, peripheral T-cells are collected from DHF patients and stained with an MHC tetramer complexed with a dengue-specific peptide. Tetramer positive T-cells can then be isolated and examined. When the tetramer positive cells were stained with Ki67, they show definitive proliferation. The cells are also found to be 'massively' apoptotic as determined by TUNEL staining. The balance of apoptotic cells with proliferative cells may skew T cell responses toward a cross-reactive phenotype. When looking at the specific T-cells involved in secondary infections with DENV1, many of the T-cells show a preference for DENV3 tetramers and infections with DENV2 show preferences in T-cells for DENV1 and 3. Clearly, viruses are able to stimulate a variety of cross-reactive T-cell responses. Memory T-cells have a lower activation threshold than do naïve T-cells[37] and the low affinity non-neutralizing cells are potentially less efficient in clearing the virus[38]. Using a similar approach in a patient currently infected with DENV1 and a previous infection of DENV2, scientists find that 21% of the T-cell population reacted preferentially to DENV2 and 11% were specific for DENV1[39]. Sixty-eight percent of the T-cells in that study were fully cross-reactive between DENV2 and DENV1. When these cells are stimulated with DENV1 derived peptides, 51% of the cells specific for DENV2 responded with either granulation or cytokine release (TNFα/IFNγ) and 75-80% of the cross reactive and DENV1 specific T-cells responded to DENV1 epitopes. The role for the 49% of cells that demonstrate low affinity for DENV1 and do not respond to peptide stimulation is currently unknown, though their proliferation is certainly suggestive. When scientists infect immune competent mice with low dose heterologus dengue viruses they find enhanced CD8+ T-cell responses that were dependent on sequential viral infection as opposed to antibody enhancement. Enhanced cell mediated immunity likely causes target cell lysis through Perforin while bystander cell death is mediated through Fas ligand binding[40].

There is a differential cytokine secretion in response to antigen exposure in CD4+ cells in Dengue infected donors. The highest IFNγ response was seen when cells were exposed homologous antigen. The cross-reaction IFNγ response could potential confer limited protection, however, when cells were exposed to heterologus antigens they produced significantly higher amounts of TNFα in relation to IFNγ[41]. During primary infections in mice, dengue specific CD4+ cells were low; however, in all four viral serotypes of a secondary infection there is a marked increase CD4+ response. Not only did CD4+ cells increase IFNγ production, but they increased CD8+ effector cell activation[42].

### Autoimmune disorder

In addition to the antibody enhancement and T-cell disruption, autoimmune disorders are also important to dengue pathogenesis. Anti-NS1 antibody responses in mice have been shown to be cross-reactive in a variety of tissues. When human anti-NS1 antibodies were developed they showed affinity for human fibrinogen, thrombocytes, and endothelial cells[43]. NS1 is a glycoprotein that is secreted by infected cells, heavily present in patient serum supernatants, lacks a membrane spanning motif, but is not, itself, present in the virus. NS1 is known to be a major immune target and high concentrations of anti-NS1 antibodies have been found in severe disease in patient studies[44]. When cells are exposed to NS1 antibodies they undergo intrinsic apoptosis, characterized by DNA fragmentation and phosphatidylserine exposure. Bcl-2 and Bcl-x decreased and P53, Bax, and cytochrome c increased in an iNOS dependent fashion. Apoptosis in response to Anti-NS1 antibody treatment can be blocked with an iNOS inhibitor[45]. The antibody mimicry is intensely inflammatory with anti-NS1 antibodies stimulating the release of IL-6, IL-8, and MCP-1 in an NFκB-dependent manner. Correlated with antibody binding is the upregulation of ICAM1. ICAM1 upregulation can facilitate the adherence of PBMCs to the endothelium. Both NFκB inhibitors and soluble NS1 to block the anti-NS1 antibodies can able to block cytokine release *in vitro*[46]. Using ELISA flow cytometry, it can be shown that NS1 binds to the surface of uninfected cells. NS1-binding motifs are commonly found in heparan sulfate and chondroitin sulfate E. In mouse experiments, tissues with a preponderance of these proteins are especially susceptible to this interaction and NS1 can be found bound to cells in the lung and liver but not intestine or brain endothelium of mouse tissues[47]. There is a high correlation between NS1 concentration in patient sera and high concentrations of anaphylatoxins which suggests a role for NS1 in complement activation. Further, anaphylatoxins are co-localized to the lungs and plasma in dengue infections. Co-localization experiments with membrane bound NS1 and NS1 specific antibodies showed the formation of complement attack complexes. Fluid phase NS1 can independently activate complement. Supernatants collected from dengue infected cells and mixed with normal sera shows complement activation by NS1 in a dose dependent manner[48]. When Dengue infected supernatants are mixed with purified antibodies from the sera from convalescent patients infected with dengue, complement activation is greatly enhanced. When they added purified NS1 protein to normal or convalescent sera they found synonymous results with NS1 activating complement and complement activation being synergized by anti-dengue antibodies. While NS1 could clearly activate complement in the fluid phase it was unable activate complement when stably expressed on the surface of cells. However, when patient samples were analyzed the found a strong correlation between NS1 concentration and C5b-C9 complex formation.

## Complement activated by dengue protein and antibodies

The complement pathway is an ancient defense mechanism designed to serve both in a sentinel capacity and in effector function. There are three pathways to complement activation. The classical pathway begins with the formation of an antibody C1q complex on the surface of a pathogen or pathogen infected cell. This complex, in turn, activates C2 via serine proteases and is itself also a serine protease[49]. The protein C2a combines with newly cleaved protein C4a to generate a C3 convertase, C2aC4b. C3b forms the central protein complex of the complement system either by binding to complement receptors or by complexing with C2aC4b to form C5 convertase, C2aC4bC3b. This complex can bind and stabilize C5a that forms the central effector function of the complement system around which proteins C5-C9 will bind and cooperatively lyse the cell. The mannose binding pathway has a similar cascade as the classical pathway but functions independently of antibody formation. Instead, MASP1 and MASP2 bind to mannose binds to the mannose structures commonly found on pathogens. The Mannan-binding lectin complex is closely homologous to C1q and can activate C2 and C4[50]. In the absence of sialic acid sugars present on normal somatic cells and which are rare on pathogens, C1q begins a lytic cascade. There is a third pathway for complement activation that begins with spontaneous activation of complement proteins. In this pathway the thioester bonds in C3 undergo hydrolysis which allows the binding of Factor B and its subsequent cleavage by plasma protease Factor D. C3b and Factor Bb combine to form a C5 convertase. Runaway complement activation is prevented by binding of Complement Receptor 1 (CR1) and a constitutively active membrane bound Decay Accelerating Factor (DAF, or CD55) which can prevent the complement cascade[51]. In patients with severe dengue, large amounts of C3a have been detected revealing a role for complement in dengue pathogenesis. This finding might be anticipated by the immune complexes that are the putative mechanism for dengue hemorrhage and shock syndromes. C3a finds some measure of importance by being one of several anaphylatoxins produced by complement activation capable of disrupting vasculature. C3a serves to recruit monocytes, macrophages, and dendritic cells, regulates vasodilatation, and increases permeability of small blood vessels and smooth muscle contraction. In macrophages, eosinophiles, and neutrophils anaphylatoxins can induce oxidative burst, basophiles, and mast cells release histamine, and C3a can enhance the effect of other proinflammatory cytokines such as TNFα, IL-6, and SDF-1. While the mechanism for the many reactions precipitated by complement anaphylatoxins has not been fully elucidated, activation of C3aR promotes cytokine expression through AKT phosphorylation as well as MAP kinase activation. C3aR is expressed on key mediators of the immune system like neutrophils, basophiles, eosinophiles, mast cells, monocytes/macrophages, dendritic cells, microglia, as well as, non myeloid cells like astrocytes, epithelial cells, smooth muscles cells, and activated T-cells, but, interestingly, not naïve T-cells. C5aR also activates a number of downstream signaling pathways including PI3K-γ (Phosophoinosital -3 Kinase), PLC (Phospholipase C), PLD (Phospholipase D), Raf and WASP (Wiskott-Aldrich syndrome protein). As a key modulator of the immune system, complement derived proteins clearly have the capacity to affect an extraordinarily large number of cell types and tissues.

### Anaphylatoxins

While TNF secretion and immune cell recruitment might be appropriately devastating, the effects of anaphylatoxins (AT) can be equally profound. C3a and C5a regulate vasodilatation, increase permeability of blood vessels, and can trigger degranulation and oxidative burst from neutrophils, eosinophiles, and basophiles. C3a and C5a act on specific receptors to produce local inflammatory responses and when secreted in concentrations high enough to invoke a general systemic response, they cause circulatory collapse similar to an IgE mediated allergic response. ATs modulate the secretion of IL-6, and TNFα from B cells and serve as potent chemoattractants[52]. C5a also works directly on neutrophils and monocytes to increase adhesion molecules, migration, and phagocytosis. Some of the primary sources of C3 are APCs such as dendritic cells and macrophages. Following antigenic stimulation both DCs and T-cells upregulate C3a and C5a receptors, produce C3, alternative complement factors B and D, and downregulate CD55. Reduced CD55 promotes T-cell proliferation and Th1 cytokine expression. In addition to C3 production, APCs cleave C3 leading to autocrine and paracrine C3R signaling. C3R signaling promotes MHC class II expression, IL-12 production and B7 co-stimulatory molecules. Dendritic cells that fail to express C3aR suffer reduced T-cell activation. Anaphylatoxins are well known initiators of inflammation but their role in regulating APC-T cell interactions is becoming increasingly prominent as more information is published. The crux of dengue pathogenesis lies in misregulation of immune processes and complement is sitting, figuratively, at the center of multiple key pathways. Anaphylatoxins become activated by DC antigen uptake and presentation. Cross reactive antibodies activate complement still further. The increase in alternative complement proteins, complement receptors and C protein all facilitate a positive feedback loop that can have dangerous consequences in a dengue infected patient.

## Conclusion

Three immune components interact to produce a confluence of symptoms that define DHF/DSS. Dengue virus initially infects immature dendritic cells through the mediation of DC-SIGN. Infected dendritic cells contribute to pathogenesis through production of metalloproteases and cytokines. Downstream of dendritic cells T-cells become activated and generate the very cytokines implicated in vascular leak and shock in addition to activating effector cells. Antibody enhancement is mediated by Fc receptors which are prominently on mature dendritic cells. Viral replication mediated by antibodies is enhanced 100-fold. In addition their effects on dengue replication, antibodies to viral epitopes cross react with cell a protein which has the effect of stimulating CD8 effector cells and production of cytokines and anaphylatoxins. Anaphylatoxins can be generated directly through viral proteins or through formation of an antibody-complement complex. Anaphylatoxins in turn can alter the reactivity of T-cells. Each year 50 million people will acquire dengue fever; 2.5 billion people are at risk. There are few components of the immune that are unaffected by the virus. There are yet questions unanswered and the virus continues to spread unabated. However these immune components are several key elements attractive targets for study that hopefully can advance the field of research.

## Competing interests

The author declares that they have no competing interests.

## Authors' informations

David Gentry Nielsen was born 27, September 1982 in Reno Nevada. Shortly afterwards he and his family relocated to Brewster Washington. David attended Andrews University where he majored in Biology with a molecular emphasis and minored in Chemistry. He joined the department of Biomedical Sciences in the Tulane School of Medicine in 2005. During the "Hurricane Semester" he accepted a gracious invitation to the University of Washington in order to continue his studies while New Orleans recovered and returned to Tulane in 2006. He completed his Master's degree with this thesis in 2009.

## Acknowledgements

I would like to acknowledge Dr. Robert Garry, Dr. Cindy Morris, Dr. Tom Voss, Dr. Deborah Sullivan, and Dr. Wimley for their personal and scientific contributions to this project. Their knowledge and expertise in were critical for its completion.
